# DNA Methylation-Related circRNA_0116449 Is Involved in Lipid Peroxidation in Traumatic Brain Injury

**DOI:** 10.3389/fnmol.2022.904913

**Published:** 2022-06-02

**Authors:** Ping Zheng, Dabin Ren, Cong Yu, Xiaoxue Zhang, Yisong Zhang

**Affiliations:** ^1^Department of Neurosurgery, Shanghai Pudong New Area People’s Hospital, Shanghai, China; ^2^Department of Key Laboratory, Shanghai Pudong New Area People’s Hospital, Shanghai, China

**Keywords:** DNA methylation, circ_0114669, lipid peroxidation, nuclear receptor, traumatic brain injury, NR1D1, NR1D2

## Abstract

Circular ribonucleic acid (circRNA) has a critical effect in central nervous diseases; however, the exact role of circRNAs in human traumatic brain injury (TBI) remains elusive. Epigenetic modifications, such as DNA methylation, can modify the mRNA level of genes without changing their related DNA sequence in response to brain insults. We hypothesized that DNA methylation-related circRNAs may be implicated in the mechanisms of TBI. The methylation-related circ_0116449 was identified from differential methylation positions and shown to reduce the neuronal loss and lipid markers. Mechanical study indicated that circ_0116449 functions as a miR-142-3p sponge and increases the expression of its target gene: NR1D2, together with NR1D1 and RORA to suppress lipid peroxidation both *in vitro* and *in vivo*. Our study suggests that DNA methylation-related circ_0116449 may be a novel target for regulating lipid metabolism in TBI.

## Introduction

Traumatic brain injury (TBI) is reported to associate with neurodegeneration, cognitive impairment, and psychiatric disorder, which has an enormous burden on the modern society (Collaborators, 2019). However, exact molecular and pathological changes in TBI are not clear. Lipid metabolism is considered to be a major pathological procedure in TBI (Piastra et al., 2020), and several targeting lipid dysregulation medicines have been applied to treat TBI patients, however, the neurological outcome is not stable (Hamdeh et al., 2021). This leads us to investigate the underlying mechanism of lipid dysregulation in TBI.

Recently, non-coding RNAs are becoming novel targets for both mechanism and therapeutic study in regulating lipid metabolism (Lu et al., 2019). However, there are few studies of non-coding RNAs in lipid dysregulation in TBI. CircRNA has a much higher expression in the brain, and it is also an independent biomarker in central nervous system (CNS) (Rybak-Wolf et al., 2015). Currently, Jiang et al. (2019) reported a series of circRNAs widely distributed in the cortex of TBI mice, however, the exact role of circRNAs in lipid metabolism in human TBI is not clear (Yuan et al., 2020).

Environmental factors can modify the mRNA level of genes without changing their related deoxyribonucleic acid (DNA) sequence. The epigenetic modification includes DNA methylation, his tone modifications, and non-coding RNAs (Pagiatakis et al., 2021). Epigenetic changes in TBI were previously observed in experimental TBI *in vivo* and *in vitro* (Hamdeh et al., 2021). However, no previous studies have evaluated the relationship between epigenetic modification and non-coding RNAs in human TBI.

We hypothesized that TBI may induce epigenetic alterations in genes involved in lipid metabolism. Accordingly, we analyzed the gene expression profiling in chipset data in TBI patients, and further combined analysis with DNA methylation to explore the function of DNA methylation related genes. We identified a new methylation related TF-circRNA-mRNA axis might be involved in the lipid peroxidation in TBI.

## Materials and Methods

### Sample Collection

Peripheral human whole blood was prospectively obtained and transferred into PAX RNA Tubes (BD, China) within 1 day post TBI (Ren et al., 2020). TBI patients were recruited based on their initial head computed tomography (CT) findings, which demonstrated brain contusions. The study protocol was approved by the Local Ethics Committee in Shanghai Pudong New area People’s Hospital (20170223-001 on 7th March 2017, updated on March 1, 2021 with a new No. K02). TBI patients were classified according to the Glasgow coma scale (GCS) score: severe group (GCS 3–8), moderate group (GCS 9–12), and mild group (GCS 13–15). We included patients with 18–65 years old, with a closed brain injury. We excluded patients with the following points: (1) Severe complication with thoracic or abdominal injury (2) Serious previous diseases (such as thrombocytopenia and cancer). (3) The family refused to undergo the blood collection. The informed consent for participating in the project was obtained from the family members and healthy controls. Clinical information for patients is listed in Table 1.

**TABLE 1:** Clinical characteristic of patients with traumatic brain injury.

Patient	Severity	Age (year)	Sex	Brain injury	GCS	Injury Type	Time post injury	Previous history	GCS (EVM)
Mi 1	Mild	30–60	F	Laceration	14	Traffic	1 h	No	E3V5M6
Mi 2	Mild	< 30	M	Contusion	15	Drop	2 h	No	E4V5M6
Mi3	Mild	30–60	M	SAH	15	Drop	1 h	No	E4V5M6
Mi4	Mild	30–60	F	SDH	15	Traffic	2 h	No	E4V5M6
Mo1	Moderate	< 30	F	Laceration	12	Drop	2 h	No	E3V4M5
Mo2	Moderate	30–60	F	SDH	12	Traffic	1 h	No	E3V3M6
Mo3	Moderate	< 30	M	SAH	12	Traffic	4 h	No	E3V4M5
Mo4	Moderate	30–60	M	SAH	12	Drop	1 h	No	E2V4M6
Se1	Severe	< 30	M	Brain stem	3	Drop	4 h	No	E1V1M1
Se2	Severe	30–60	F	Contusion	8	Traffic	3 h	No	E2V2M4
Se3	Severe	30–60	F	SDH	6	Drop	1 h	No	E1V1M4
Se4	Severe	30–60	M	SDH	6	Traffic	2 h	No	E1V1M4
Co1	Control	30–60	M						
Co2	Control	30–60	F						
Co3	Control	30–60	F						
Co4	Control	30–60	M						

*SAH, subarachnoid hemorrhage; SDH, subdural hemorrhage.*

### Microarray Information

The Agilent Human lncRNA Microarray 2019 (4*180k, Design ID: 086188) was used in this experiment and data analysis of the 16 samples were conducted by OE Biotechnology Co., Ltd. (Shanghai, China).

### Gene Microarray

Total RNA was quantified by the NanoDrop ND-2000 (Thermo Scientific, CA, United States) and the RNA integrity was assessed using Agilent Bioanalyzer 2100 (Agilent Technologies, CA, United States). The blood RNA manipulation was according to the manufacturer’s protocols. In brief, total RNA was transcribed to cDNA, and synthesized into cRNA and labeled with Cyanine-3-CTP. The labeled cRNAs were hybridized onto the microarray chipset. After being washed, the chipset was scanned by the Agilent Scanner G2505C (Agilent Technologies, CA, United States).

### Sc-RNA-seq Analysis

The single-cell transcriptome dataset GSE160763 was downloaded from the Gene Expression Omnibus (GEO) database (Witcher et al., 2021). The chipset data from patients with TBI were previously reported and used as an external verification here (Ren et al., 2020).

The Seurat package was used for the sc-RNA seq study (Hao et al., 2020). The dimension of data was reduced by principal component analysis (PCA) and *t*-distributed stochastic neighbor embedding (t-SNE). Marker genes for different clusters were identified using the Seurat package. All clusters were annotated using the SingleR package with a mouse dataset (Aran et al., 2019) and TF analysis was performed using the SCENIC package.

### DNA Methylation Preprocessing

DNA methylation chipset data were obtained from the public dataset (GSE 155426), where patients with TBI were classified into mild and severe group. The annotation of the investigated probes was based on the 850K Chip array to test TBI patients’ blood. Cytosine-phosphate-guanine (CpG) markers present on EPIC chipset were classified according to their chromosome location, and the feature category gene region as per UCSC annotation (TSS200, TSS1500, 5′UTR, 1st Exon).

### Differentially Methylated Loci Analysis

All bioinformatical analysis was performed with the R (version 4.1.0). Original IDAT files were processed with the Minfi package (version 1.28.3) and DMPs were located through ChAMP package (Aryee et al., 2014; Tian et al., 2017). Based on Illumina’s manifest, DMPs were assigned to genes and candidate genes were identified based on a nominal *p*-value cutoff < 1e-4 and 10 or more DMPs per gene. Significant DMPs were mapped to genes based on cg CpG sites, and candidate genes were selected based on *p*-value cutoff < 0.01.

### Functional Network Analysis and Visualization

For functional studies, we corroborated these enriched pathways using cluster Profiler R package (4.0.2), which summarizes and visualizes lists of GO terms. Specifically, cluster Profiler was used to cluster significantly enriched GO terms (FDR < 0.05) into similar functions (Wu et al., 2021).

### CeRNA Network Construction

First, we showed the circRNA is mainly located in the cytoplasm. As most cytoplasmic circRNA acts as a miRNA sponge, we checked a series of miRNAs that circ_0114669 might bind to with an online tool (CircInteractome) and found only miR-142-3p was previously reported to increase in TBI session (Panda et al., 2018). We further predicted the target genes of miR-142-3p by integrating the results from TargetScan (Agarwal et al., 2015), miRDB (Chen and Wang, 2019) and TargetMiner (Chen Y. A. et al., 2019).

### Primary Cortical Neuronal Cultures

Primary culture of cortical neurons was previously reported (Chen W. et al., 2019). For pharmaceutical intervention, cells were given Circ_0116449, miR-142-3p, or si-Nr1d2 and incubated together with culturing medium.

### Mouse Traumatic Brain Injury Model and Lipid Metabolism Markers

The lateral FPI surgery was performed in 6–8 week-old male C57-B6 mice as previously described (Chen W. et al., 2019). Relative expression was calculated with the formula: 2^–ΔΔ*Ct*^, ΔΔCt = (Ct_target gene_ − Ct_β –actin_) TBI − (Ct_target gene_ − Ct_β –actin_) control and repeated at least three times. GAPDH was used as a circ_0116449 internal control. The circ_0116449 primers were forward, 5′- AGA GGA GTG GTG GGT TTT CC -3′ and reverse, 5′- CGC TGC TCA TAG TAA AAT CTG G -3′. The GAPDH primers were forward, 5′-TGC ACC ACC AAC TGC TTA GC-3′ and reverse, 5′-GGC ATG GAC TGT GGT CAT GAG-3′. Cellular lipid markers (CD36 and UCP3) are evaluated by qPCR in human TBI patients. Serum MDA and superoxide dismutase activity (SOD) levels were tested with ELISA in TBI mice as well.

### RNA FISH Assay

The sub-localization of circ_0116449 in neurons was identified using RNAFISH according to the instructions of RiboTM circ_0116449 FISH Probe Mix (Green; Guangzhou Ribo Biology Co., Ltd., Guangzhou, Guangdong, China). The neurons were seeded into the plate at 6 × 10^4^ cells/well and cultured until the cell confluency reached about 80%. Then, the neurons were fixed by 1 mL 4% paraformaldehyde, treated with proteinase K (2 μg/ml; Sigma-Aldrich Chemical Company, St Louis, MO, United States), glycine (YZ-140689; Beijing solarbio science and technology Co., Ltd., Beijing, China), and acetamidine reagent. The slides were then incubated with 200 μl pre-hybridization solution at 42°C for 1 h and with 200 μl 280 ng/ml hybridization solutions containing circ_0116449 probe overnight at 42°C. After that, the slides were stained for 5 min with DAPI (ab104139, 1: 100, Abcam Inc., Cambridge, United Kingdom) diluted using PBS-Tween 20. The slides were then mounted with an anti-fluorescent quencher. 3 different fields were selected under a fluorescence microscope (Olympus Optical Co., Ltd., Tokyo, Japan) for observation and photographs.

### Plasmid, siRNAs, and miRNA Mimic, Inhibitor, Transient Transfection, and Construction of Stable Cell Lines

Plasmid-mediated circRNA over expression vector were obtained from GenePharma (Suzhou, China), siRNAs targeting circRNA, and miR-142-3p mimic was ordered from RiboBio (Guangzhou, China), and control plasmid was ordered from GeneCopoeia (Rockville, MD, United States). For stable transfections, we used Puromycin to select circ_0116449 and negative vector stable expressing cells. TheCirc_0116449 or si_Nr1d2 plasmid was injected into the lateral ventricle for mice.

### CCK-8 Assay

Each group of cells was adjusted at 1,000 cells per well. About 10 μl of CCK-8 Solution (Beyotime Biotechnology) was added to the cell dish after 24 h, and blank control has only CCK-8 solution. Absorbance (OD) value of each well was read for each well at 490 nm and tested every 24 h for 3 days.

### Reactive Oxygen Species and Apoptosis Assay for FACS

The Annexin V-FITC/propidium iodide (PI) apoptosis detection kit and reactive oxygen species (ROS) detection kit were obtained from Nanjing KeyGen Biotech Co., Ltd. (Nanjing, China). The Annexin V-FITC/PI kit was used to detect the apoptosis of cells by flow cytometry. The cells were then washed, adjusted to 1 × 10^6^ cells/ml and stained with Annexin V-FITC, and PI solution for 15 min at room temperature in the dark. Finally, the stained cells were analyzed by flow cytometry (Beckman Coulter, Inc., Brea, CA, United States). For the ROS assay, the primary cultured neurons were resuspended in culture medium (without serum) containing 10 μM DCFH-DA. As positive control, cells were treated with Rosup (50 μg/ml) for 30 min. Subsequently, ROS production was detected by flow cytometry.

### Elisa Method for Serum Malondialdehyde, Superoxide Dismutase Activity and Glutathione

The level of serum malondialdehyde (MDA) was determined by colorimetric assay (Jiancheng, Nanjing, China, A003-1). After the extracted serum was added with the working solution, they would be incubated at 95°C for 60 min and cooled on ice as protocol suggested, and the MDA activity was read out by a microplate reader at 532 nm.

The activity of serum SOD was determined by colorimetric assay (ab65354). After the extracted serum was added with the working solution, they would be incubated at 37°C for 30 min as protocol suggested, and the SOD activity was read out by a microplate reader.

The level of glutathione (GSH) was tested with a reduced assay (Jiancheng, Nanjing, China, A006-2-1), where GSH can have a chemical effect with dithio-bis-nitrobenzoic acid (DTNB) to form a yellow compound which could be assessed at 405 nm by colorimetry.

### Statistical Analysis

All data are presented as the mean ± standard error mean (SEM). GraphPad Prism 8.3.1 (San Diego, CA, United States) was used for statistical analyses. Differences among more than 2 groups were analyzed using one-way ANOVA and further LSD test or Student’s *t*-test. Repeated one-way ANOVA was used to analyze results of CCK-8 assays. Spearman correlation analysis was used to assess the relationship between 2 parameters, such as Circ_0116449 and NR1D2 with GCS. *p* < 0.05 was considered significant.

## Results

### Differentially Methylated Positions in Patients With Traumatic Brain Injury

**FIGURE 1:**
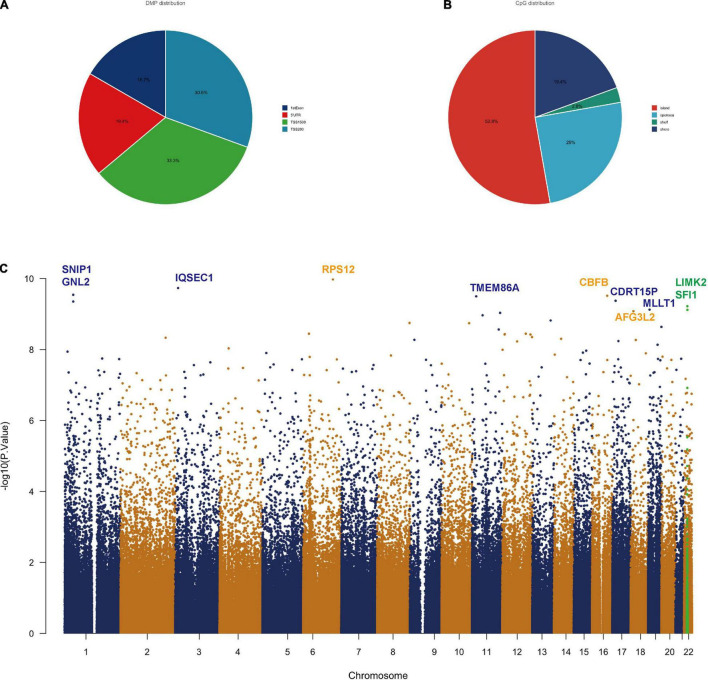
Differentially methylated position analysis in TBI patients. **(A)** Intragenic differentially methylated position (DMP) distribution: approximately 83% of the DMPs are located in the promoter. **(B)** CpG allocation: > 50% of the CpGs is in the island. **(C)** Manhattan plot showing chromosomal locations of –log10 (*p*-values) for the association at each locus. Top DMPs (log10P value > 9.0) in the methylation set are annotated by gene symbols.

We found statistically significant differences in 18,253 CpGs between severe TBIs and mild TBIs. To specifically evaluate the methylation patterns and the preferential genome location, we segmented all significant differentially methylated positions (DMPs) with a *p*-value < 1e-6 into the following four categories according to the Illumina annotations: transcription start site (TSS) 1500, TSS 200 (indicating the number of bases upstream), 5′ untranslated region (UTR), 1st exon (Figure 1A). We can see about 1/3 DMPs lied at TS1500 and TSS 200 areas. The DMP distribution within a CpG island is also shown in Figure 1B, with the majority located in the island (> 50%). Additionally, we generated a Manhattan plot to observe, at the chromosomal level, where the significant DMPs were located. The results of this analysis indicated that all of the DMPs were evenly distributed throughout the chromosomal complement (Figure 1C). Those with a very high *P* value of DMPs were listed in Table 2.

**TABLE 2:** A list of genome-wide significant DMPs with log10P value > 9.0.

CpG site	*p*-value	Q value	CHR	Gene symbol	SNP	FC	log10P value
cg16226300	1.05E-10	4.76E-05	6	RPS12	cg16226300	−0.4448408	9.97819942
cg24241688	1.85E-10	4.76E-05	3	IQSEC1	cg24241688	0.35256533	9.73204576
cg27184249	2.86E-10	4.76E-05	1	SNIP1	cg27184249	0.13631913	9.54399017
cg02709840	3.04E-10	4.76E-05	16	CBFB	cg02709840	−0.2921311	9.51724811
cg05744487	3.16E-10	4.76E-05	11	TMEM86A	cg05744487	−0.205656	9.49995018
cg00170799	4.23E-10	4.76E-05	17	CDRT15P	cg00170799	0.09314366	9.37390554
cg16441688	4.44E-10	4.76E-05	1	GNL2	cg16441688	0.16662105	9.35304908
cg24315703	6.01E-10	5.65E-05	22	LIMK2	cg24315703	−0.1754699	9.22100023
cg23084309	7.48E-10	5.68E-05	19	MLLT1	cg23084309	−0.129443	9.12585746
cg08226295	**7.62E-10**	**5.68E-05**	**22**	**SFI1**	**cg08226295**	−**0.516428**	**9.11788336**
cg20012885	8.32E-10	5.68E-05	18	AFG3L2	cg20012885	−0.2133065	9.08008298
cg08941173	9.26E-10	5.80E-05	11	PKNOX2	cg08941173	−0.1330939	9.03324458

**FIGURE 2:**
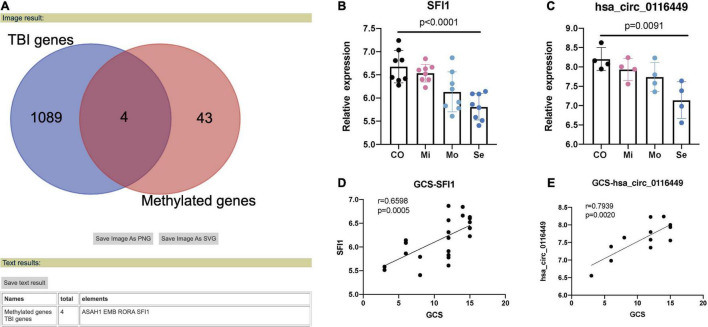
Methylated genes in TBI. **(A)** 4 methylated genes were intersected in TBI. **(B)** The expression level of SFI1 in TBI. **(C)** The expression of Circ_0116449 in TBI. **(D,E)** The correlation of GCS and expression level of SFI1 and Circ_016449 in TBI, respectively. The *p* value is obtained by the comparison among all 4 groups.

*SFI1 is selected with the bold values, which is intersected with TBI genes in Figure 2A.*

### The Expression Level of circ_0116449 in Traumatic Brain Injury

Next, we crossed the methylated genes with the DEGs in TBI (TBI samples against controls) and we got four intersected genes named: ASAH1, EMB, RORA, and SFI1 (Figure 2A). We found both the expression of RORA and SFI1 has a downward trend with the severity of TBI (Figures 2B,C). More importantly, after the co-expression analysis, we identified a new circRNA (circ-SFI1, named has_circ_0116449). Both the expression level of SFI1 and circ_0116449 decreased with the severity of TBI and is positively correlated with GCS in TBI patients (*r* = 0.6598 and 0.7939, respectively, Figures 2D,E).

### Circ_0116449– miR-142-3p –NR1D2 Axis in Traumatic Brain Injury

**FIGURE 3:**
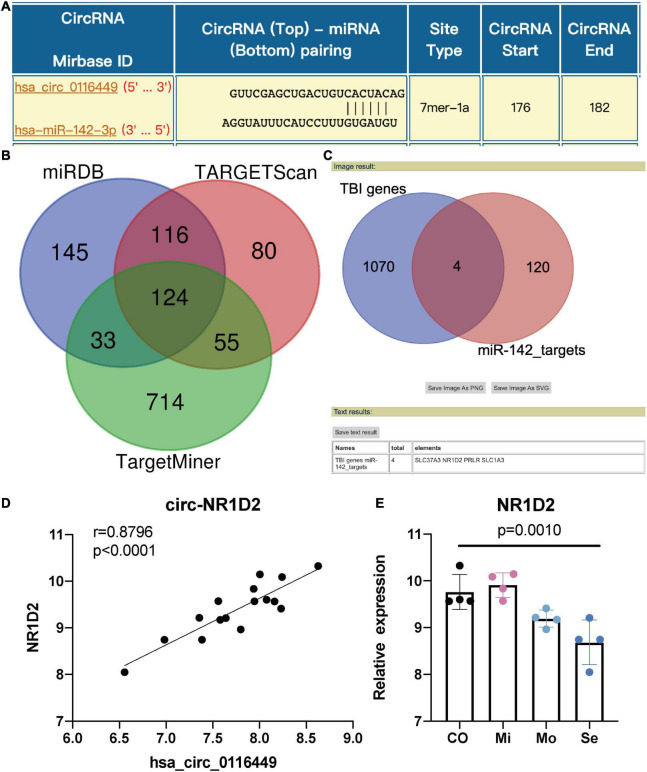
Circ_0116449 binds with miR-142-3p and NR1D2 in neurons. **(A)** Based on the interactome prediction, miR-142-3p was supposed to bind to circ_016449; **(B)** the target genes of miR-142-3p is shown in the Venn figure; **(C)** Intersected genes between miR-142-3p targets and DEGs in TBI; **(D)** The Spearman correlation between the expression of circ_0116449 and NR1D2 in human TBI blood, *r* = 0.8796, *p* < 0.0001; **(E)** The relative expression of NR1D2 in different severity of TBI. The *p* value is obtained by the comparison among all 4 groups.

**FIGURE 4:**
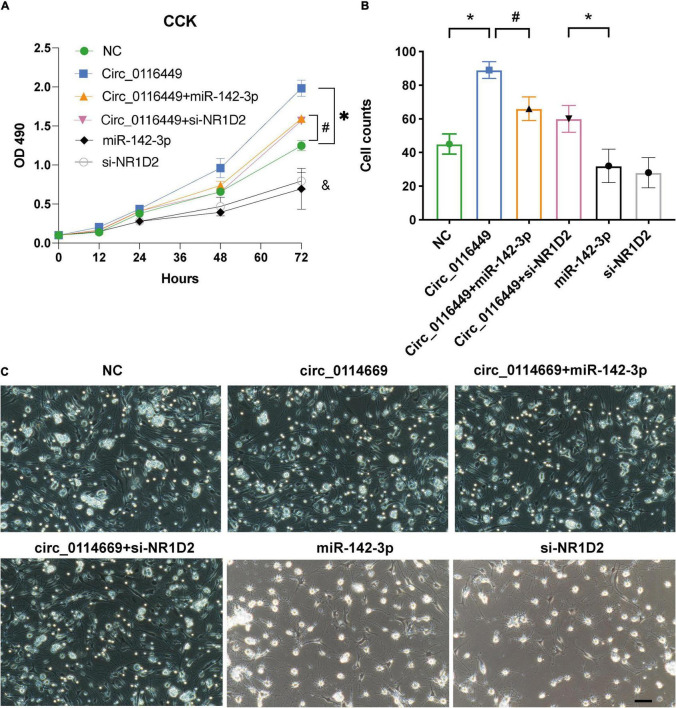
Circ_0116449 on neuronal proliferation. **(A,B)** After primary cultured neurons were transfected with NC, circ_0116449, circ_0116449 + miR-142-3p, circ_0116449 + siNR1D2, miR-142-3p and siNR1D2, OD490 and cell counts were assessed. **(C)** The representative images of primary cultures in different groups. *, #, & (compared to the NC group) p < 0.05.

As circRNA may function as a miRNA sponge, we did a search on the CircInteractome website and found it could bind to several miRNAs. Among them, we chose circ_0116449 might bind to miR-142-3p, as miR-142-3p was previously reported to increase in TBI (Weisz et al., 2020; Figure 3A). We next determined the target genes of miR-142-3p target genes. We crossed the predicted results from miRDB, TARGETScan, and TargetMinerA, and obtained 124 shared target genes. Again, after crossing with the DEGs in TBI, we got 4 intersected genes: SLC37A3, NR1D2, PRLR, SLC1A3 (Figures 3B,C). Again, only NR1D2 has a positive correlation with the expression of circ_0116449 and downward trend with the severity of TBI, which indicated that NR1D2 was the predicted target of miR-142-3p (Figures 3D,E). To investigate the miR-142-3p target genes in neurons and their correlation with circ_0116449, cortical neurons were transfected with NC mimics, circ_0116449, miR-142-3p mimics, or si-NR1D2, co-transfected with circ_0116449 and miR-142-3p mimics, co-transfected with circ_0116449 and si-NR1D2. The data show that circ_0116449 was able to promote neuronal proliferation in CCK8 test, while this effect would be blocked by miR-142-3p mimics or reduced NR1D2 (Figure 4A). Similarly, the counts of primary cultured neurons were increased with Circ_0116449, and abolished by miR-142-3p mimics or si-NR1D2, respectively. More importantly, cell proliferation was statistically reduced in the miR-142-3p mimics or si-NR1D2 groups versus the NC group (Figures 4B,C). These results indicate that circ_0116449 promotes cell proliferation *via* miR-142-3p and NR1D2.

### GO and KEGG Analyses

**FIGURE 5:**
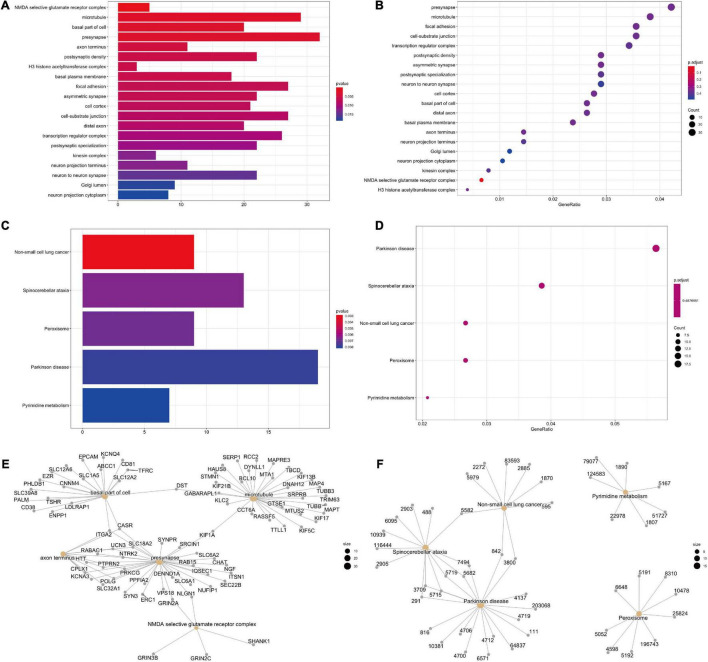
GO and KEGG pathways in DNA methylation related genes in TBI. **(A,B)** GO analysis results. **(C,D)** KEGG enriched pathways. **(E,F)** The exact genes for GO and KEGG pathways.

GO analysis of host genes of DNA methylation was carried out to show the alterations in molecular functions (MF), biological processes (BP), and cellular components (CC). We identified the methylated genes in TBI were mainly enriched in microtubule, presynapse, focal adhesion, cell-substrate junction, and transcription regulator complex (Figures 5A,B).

Then, we performed the KEGG analysis and bubble chart to show top 5 selected pathways clustered in DNA methylated genes, such as non-small cell lung cancer, spinocerebellar ataxia, peroxisome, Parkinson’s disease, and pyrimidine metabolism (Figures 5C,D). Completely, these methylated genes are mostly involved in synapse and peroxisome. The exact genes for GO and KEGG pathways are shown as well (Figures 5E,F).

### Circ_0116449 and NR1D2 Are Involved in Lipid Metabolism in Traumatic Brain Injury

**FIGURE 6:**
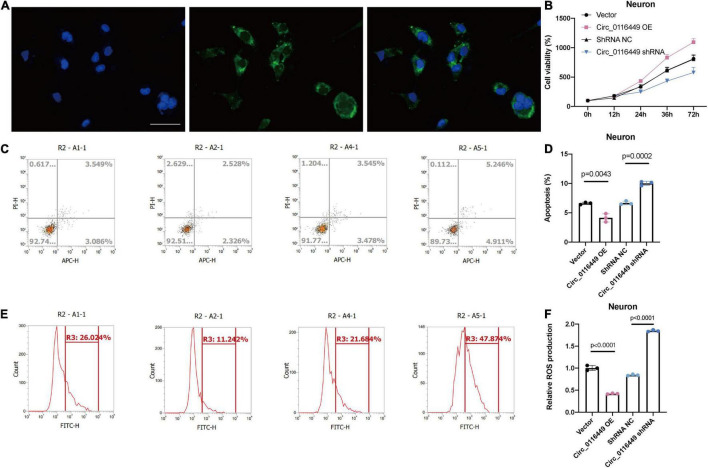
Circ_0116449 on neuronal lipid metabolism *in vitro*. **(A)** FISH results show circ_0116449 is mainly expressed in the cytoplasm. **(B)** Cell viability is affected by circ_0116449. **(C–F)** Apoptosis rate and ROS production level in different groups. Circ_0116449 reduced while si-circ_0116449 increased the apoptosis rate and ROS production in neurons. Scale bar = 150 mm.

First, we investigated the role of circ_0116449 on lipid metabolism *in vitro* and found it is mainly distributed in the cytoplasm (Figure 6A). And overexpressed circ_0116449 was able to reduce the apoptosis rate and ROS production, while knockdown of circ_0116449 could increase the apoptosis rate and ROS production in primary cultured neurons (Figures 6B,C).

**FIGURE 7:**
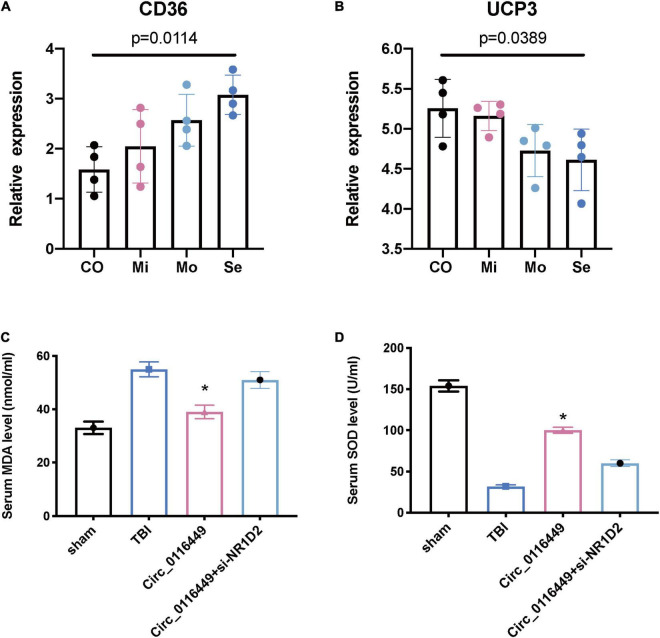
Lipid metabolism biomarkers in human patients and animal models of TBI. **(A,B)** CD36 and UCP3 relative expression in different severity of human TBI patients; **(C,D)** Serum MDA and SOD levels in sham or TBI mice treated with circ_0116449 and circ_0116449 + siNR1D2. The *p* value is obtained by the comparison among all 4 groups. The * indicates the comparison to the other groups.

Next, we further investigated the lipid metabolism in TBI. First, we found blood CD36 expression is increased with the severity of TBI (Figure 7A) and UCP3 level is decreased with the TBI severity (Figure 7B). This indicates that TBI causes lipid peroxidation. As NR1D1, NR1D2, and RORA belong to the Nuclear Receptor transcription pathway, they have a critical role in circadian rhythm, as well as lipid homeostasis and lipid metabolism (Supplementary Figure 1). As circ_0116449 is conserved well between mice and human (Supplementary Figure 2), we further investigated the role of circ_0116449 on the lipid markers in TBI mice and found the blood MDA increased and SOD level decreased in TBI mice, while circ_0116449 could reverse this pathology, and this effect could be partly blocked by knockdown of NR1D2 (Figures 7C,D). These results show that circ_0116449-NR1D2 have a role in regulating lipid metabolism in TBI.

### The Gene Regulatory Networks in Traumatic Brain Injury

**FIGURE 8:**
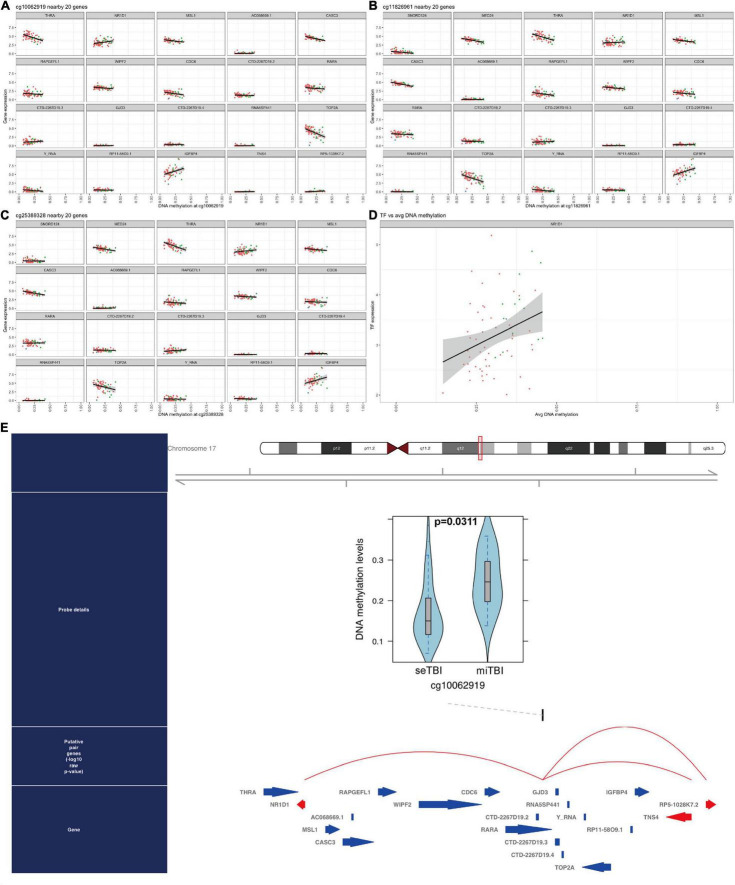
DNA methylated TFs in TBI. **(A–C)** 3 interesting CpG sites and related genes are listed: NR1D1 is included. **(D)** The linear correlation between TFs and average DNA methylation. **(E)** The DNA methylation level in cg10062919 between severe TBI and mild TBI group and NR1D1 is related to DNA methylation in this CpG site.

Finally, to investigate gene regulatory networks (GRNs) and related master regulator transcription factors, we performed ELMER analysis with 134,014 distal probes. Subsequent motif enrichment analysis identified motifs associated with the 206 probes, and these motifs were used to identify upstream TFs. Among them, we found the expression of NR1D1 (ENSG00000126368) is associated with three CpG sites: cg10062919, cg11826961, and cg25389328 (*p* = 0.03112874, Figures 8A–C). The general TFs expression is positively associated with average DNA methylation level (Figure 8D). Furthermore, we showed that DNA methylation level (β-values) at cg10062919 in severe TBI was reduced compared to mild TBI group. And this CpG is associated with several TFs as listed; the red arrow of NR1D1 indicated that this was a backward strand (Figure 8E).

### DNA Methylation Related TFs in Traumatic Brain Injury

**FIGURE 9:**
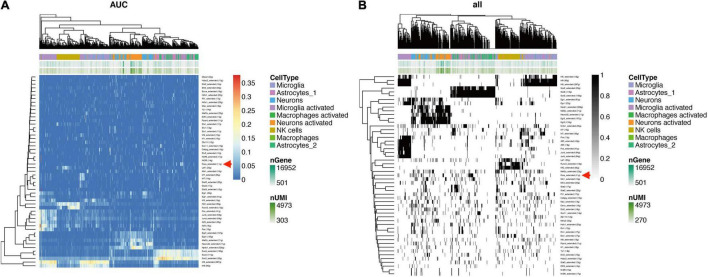
SCENIC analysis shows TFs in TBI mice in different cell clusters from GSE **(A)**. Rora_extended_11g is listed in both AUC and binary regulons **(B)**.

**FIGURE 10:**
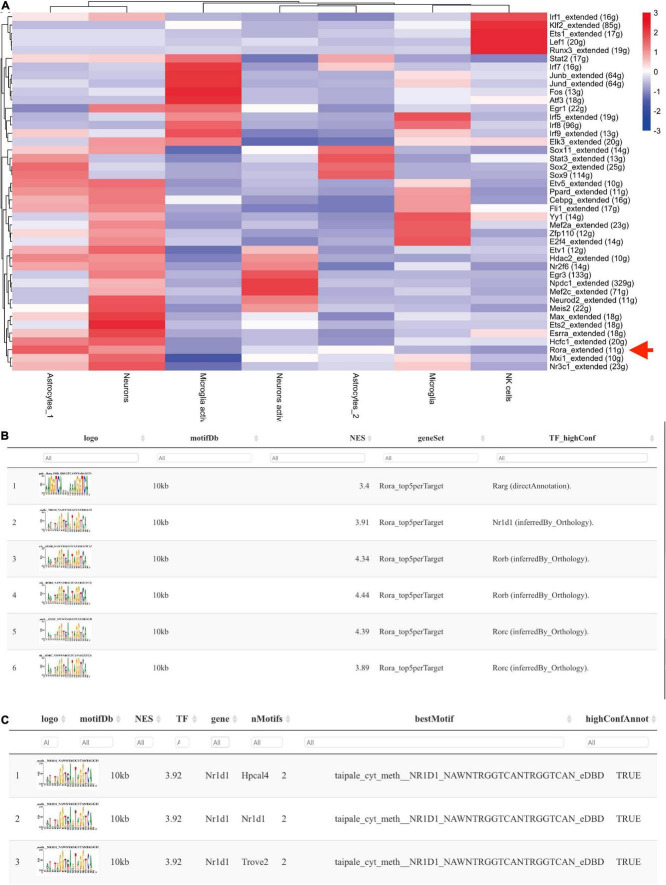
Nr1d1 and Rora with best motifs in TBI. **(A)** The heat map shows the relative expression of different TFs based on potential cell clusters in TBI; **(B,C)** The best motifs for Rora and Nr1d1 in TBI, and Rora is able to regulate Nr1d1.

To confirm the TF profiling in TBI, we did the SCENIC analysis with the sc-seq data from GSE. We identified 53 TF regulons in 9 cell clusters, and we found Rora_extended_11g is on the list in both AUC and Binary regulons (Figure 9). This means Rora is a major TF in TBI. To investigate the source of Rora, we used the heat map to show the relative expression of different TFs in TBI and found the expression of Rora is increased in astrocytes and neurons, especially in astrocytes_1 and not astrocytes_2, compared to other cell types (Figure 10A). This indicates that Rora might be an activity-dependent marker for astrocytes and neurons. Furthermore, we showed the best motifs for Rora and found it is associated with Nr1d1 (Figure 10B). And Nr1d1 has three best motifs as well, which is consistent with the DNA methylation results in TBI (Figure 10C).

## Discussion

In this study, we analyzed the blood gene profiling from patients with TBI and identified methylation-related circRNA, which is related to lipid metabolism processes. In addition, SCENIC analysis revealed differential DNA methylation-related TFs in patients with TBI in a number of genes representing biological mechanisms such as peroxisomes. Previously, there have been very few studies regarding DNA methylation in human TBI. In the current study, we found most methylated regions were at promoter area (TSS1500, TSS200 and 5′UTR) and these methylations occur at the island area (more than 50%).

The Manhattan plot showed genes with a statistically different DMP and we identified SFI1, which is a parent gene of circRNA_0116449 among them. Both the gene expression level of SFI1 and circ_0116449 decreased with the severity of TBI and positively correlated with GCS score in patients with TBI. The expression of circ_0116449 was remarkably lower after brain insults *in vitro* and *in vivo*, and its down regulation was positively correlated with SFI1 expression and GCS score in TBI. In addition, we investigated the function of circ_0116449 in neurons and found that knockdown or over expression of circ_0116449 significantly reduced or facilitated the cellular proliferation in primary cultured neurons by CCK8 assay. Additionally, circ_0116449 can reduce the cellular apoptosis and ROS production in neurons as well. Particularly, we also demonstrated mechanistically that circ_0116449 promotes the neuronal proliferation by sponging miR-142-3p to increase the expression of NR1D2. Considering the stable circular structure and enrichment in CNS, circ_0116449 may become a potential target for TBI.

GO analysis of host genes of DNA methylation showed that methylated genes in TBI were mainly enriched in microtubule, pre-synapse, focal adhesion, cell-substrate junction, and transcription regulator complex; while KEGG analysis demonstrated top 5 selected pathways were non-small cell lung cancer, spinocerebellar ataxia, peroxisome, Parkinson’s disease, and pyrimidine metabolism. These results suggest DNA methylation-related genes are critically involved in synapse and peroxisomes in TBI.

Although the roles of lipid metabolism and targeted therapy have been explored in TBI for a while, the neurological outcome is not stable from these therapies (Hamdeh et al., 2021). To understand the upstream of lipid metabolism in TBI, we integrated SCENIC based on sc-seq data and ELMER package to investigate the TF-cg-RNAs as genetic regular network in TBI and identified 206 hub probes out of 134,014 distal probes. We found the expression of NR1D1 (ENSG00000126368) is associated with 3 CpG sites: cg10062919, cg11826961, and cg25389328, and DNA methylation level (β-values) at cg10062919 in severe TBI was reduced compared to mild TBI group, which is consistent with the previous studies (Liu et al., 2022). Liu et al. found decreased DNA methylation CpG site cg22111818 in repulsive guidance molecule A (RGMA) is associated with intracranial hypertension after severe TBI. It is generally considered that reduced DNA methylation in TBI might be involved in the pathological mechanisms and higher DNA methylation is proposed to promote recovery in severe TBI (Treble-Barna et al., 2021).

In our study, we identified 53 TF regulons in 9 cell clusters with SCENIC analysis and found Rora_extended_11g is in the list in both AUC and Binary regulons. RORA, NR1D1 and NR1D2 belong to the nuclear receptor family and act as a regulator for lipid homeostasis. As lipid peroxidation occurs in TBI, based on our chipset results, we found the expression of RORA, NR1D1, and NR1D2 was all decreased in human TBI blood (Supplementary Figure 1). As very few studies have explored the mechanism of the peroxidation in TBI, our findings indicate that impaired nuclear receptor family members might contribute to the dysregulated lipid metabolism in TBI.

*In vivo* studies show that the knockdown of Nr1d1 (REV-ERBα) could promote lipid storage and insulin sensitivity (Dyar et al., 2018), and after knockout of REV-ERBα leads to pathway perturbations in fatty acid, triglyceride, and phospholipid metabolism (Dyar et al., 2014). It has also been reported that down-regulated Nr1d1 was associated with activated nuclear factor kappa B (NFκB) signaling (Welch et al., 2020), which has been previously reported by our group (He et al., 2021). Furthermore, Nr1d1 binding at positive control lock at Ucp3 was reduced in Nr1d1 knockout muscles (Welch et al., 2020). In a recent study, Ann et al. found that global Nr1d1 deletion driving dysregulation of white adipose tissue (WAT) lipogenesis and obesity (Hunter et al., 2021).

It is previously reported that Nr1d1, as a co-TF could transcriptionally regulate the expression of Connexin (Cx) 43 (Negoro et al., 2012), and we also reported that Cx is related to the stress and lipid metabolism after brain insults (Chen et al., 2017, 2018). Gja1 is predicted to be a target for both Nr1d1 and Nr1d2 from TRRUST web tool. Meanwhile, NR1D1 is a transcriptional target of RORA with an experimental validation (Zenri et al., 2012). Therefore, we applied the JASAPR database to assess the potential effect of Nr1d1, Nr1d2, and RORA on the transcription of Cx43. We found that RORA, Nr1d1, and Nr1d2 may potentially bind to the promoter of Cx43 with the similar sequence (- AGGTCA-, Supplementary Figure 3A), which is consistent with our SCENIC analysis results (The best motifs for Rora and Nr1d1 in TBI, Figures 10B,C). Then, we assessed the effect of circ_0116449 on the expression of ROS, MDA, and GSH, and found that knockdown of circ_0116449 could increase the production of ROS and MDA, while reduced the GSH level; and over expressed circ_0116449 was able to reverse this (Supplementary Figures 3B–E). However, the protective effect of circ_0116449 was partly blocked by siNr1d2 and Cx43 inhibitor.

Therefore, we further investigated the role of circ_0116449 on *in vivo* cellular lipid biomarkers. We first confirmed that CD36 (Cifarelli et al., 2021), a marker of lipid oxidation is increased with the severity of TBI and the expression level of UCP3 (an antioxidant marker) is decreased in human TBI (Leger et al., 2019). Next, we checked its expression in TBI mice and found the serum MDA is increased and circ_0116449 treatment could reduce the serum MDA level; while it can also increase the serum SOD level in TBI mice, and both effects were partly blocked by siNR1D2. These results indicate that circ_0116449 might be a new target for suppressing lipid peroxidation and nuclear receptor could become biomarkers for lipid metabolism in TBI.

There are some limitations need to be addressed in our study as well. First, the sample size in our study was relatively limited (12 TBI patients and 4 healthy controls). Future study needs a bigger sample size to confirm the current results and our findings here should be considered to be preliminary. Second, the spatial expression of circRNAs and nuclear receptors in patients with TBI might be different, and this situation is not further investigated here, which needs to be explored by spatial transcriptomics as well. Our cluster results show that upstream TFs and circ_016449 downstream NR1D2 are specifically related to lipid peroxidation, shedding light on the role of circ_016449, and nuclear receptor family in dysregulated lipid metabolism in TBI and they might become potential targets in clinical session. These findings indicate that circ_0116449 is able to regulate lipid markers dependent of nuclear receptor family and Cx43. However, the complexity of the GRN in TBI needs to be explored in future studies.

## Data Availability Statement

The raw data supporting the conclusions of this article will be made available by the authors, without undue reservation.

## Ethics Statement

The studies involving human participants and animal study were reviewed and approved by the Local Ethics Committee in Shanghai Pudong New Area People’s Hospital. Experiments were performed under ethical guidelines (20170223-001 on 7th March 2017, updated on 1st March 2021 with a new No. K02) and handled according to institutionally- approved procedures. The patients/participants provided their written informed consent to participate in this study.

## Author Contributions

PZ designed the whole study, did the cell (*in vitro*) work, collected peripheral blood, and did FACS study. DR and XZ performed the *in vitro* and *in vivo* experiments. CY and YZ analyzed the data and did the statistical analysis. PZ and YZ wrote the manuscript. All authors read and approved the final manuscript.

## Conflict of Interest

The authors declare that the research was conducted in the absence of any commercial or financial relationships that could be construed as a potential conflict of interest.

## Publisher’s Note

All claims expressed in this article are solely those of the authors and do not necessarily represent those of their affiliated organizations, or those of the publisher, the editors and the reviewers. Any product that may be evaluated in this article, or claim that may be made by its manufacturer, is not guaranteed or endorsed by the publisher.
