# Supplementary material for: DNA Methylation-Related circRNA_0116449 Is Involved in Lipid Peroxidation in Traumatic Brain Injury
Source: Front Mol Neurosci. 2022 Jun 2;15:904913. doi: 10.3389/fnmol.2022.904913 (PMC9201693; doi:10.3389/fnmol.2022.904913)
Supplement: Supplementary file 1 [file Data_Sheet_1.PDF]

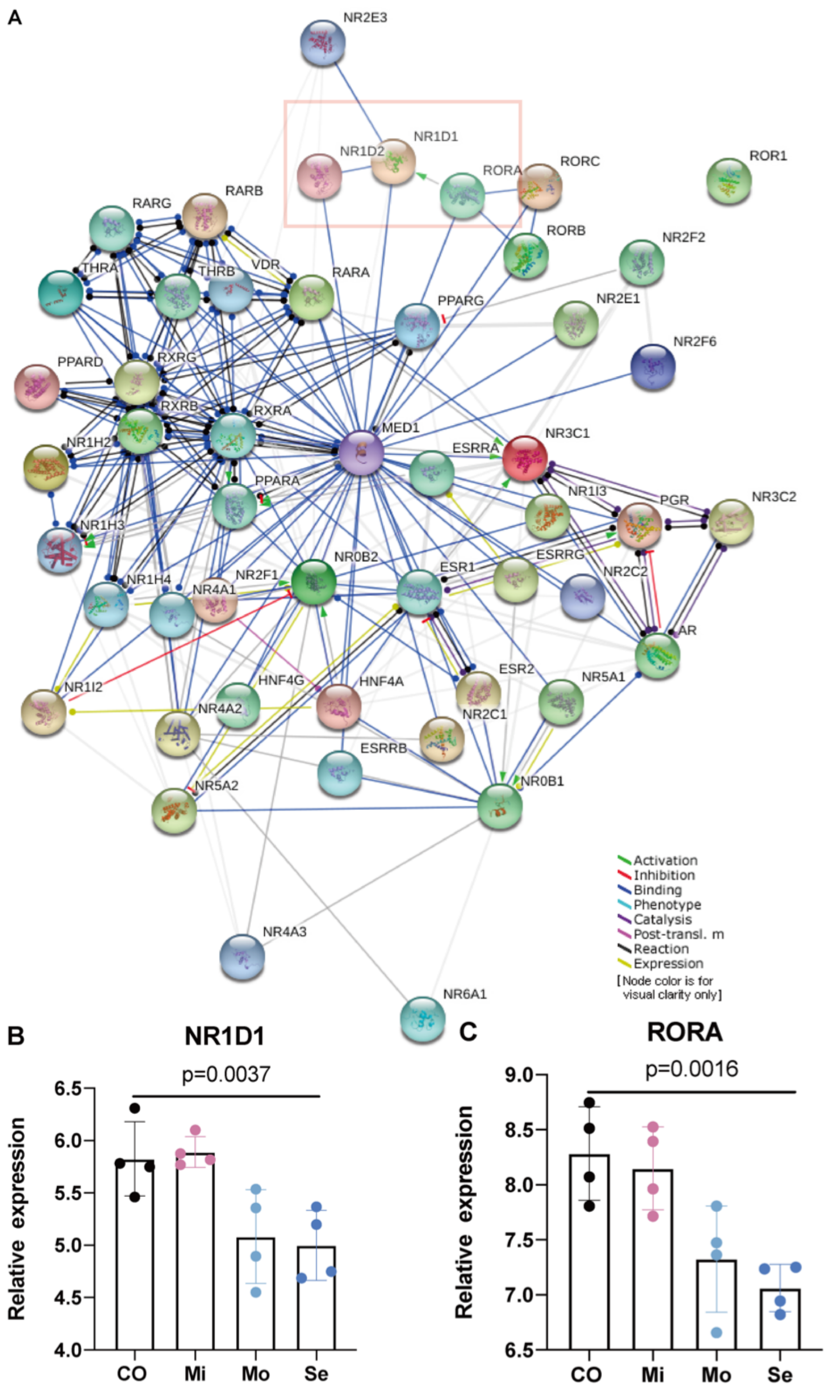

Supp Figure 1. The nuclear receptor family in TBI. (A) The network picture of nuclear receptor family. NR1D1, NR1D2 and RORA are labeled. **(B)** The expression level of NR1D1 in human TBI plasma. **(C)** The expression level of RORA in human TBI plasma. CO: Control, Mi: Mild TBI, Mo: Moderate TBI, Se: Severe TBI.

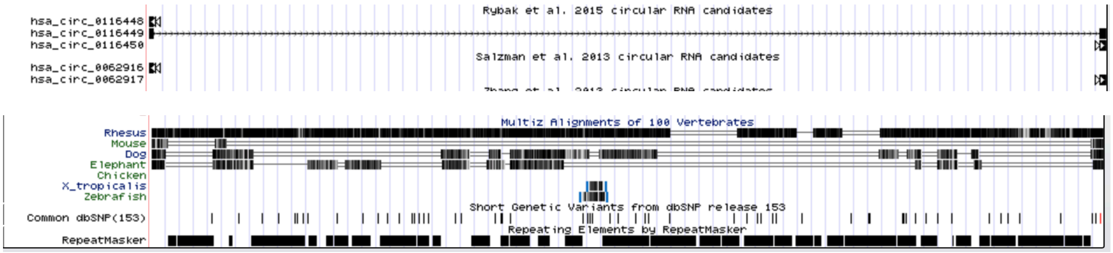

Supp Figure 2. From the UCSC list, we found only has_circ_0116449 is a full-length circRNA. And when we used the UCSC Genome Browser on Human Assembly (GRCh37/hg19) and searched the chr22:31,927,043-31,942,957, which includes 15914 bp for genomic length, we found the has_circ_0116449 was conserved in Rhesus, Mouse, Dog, Elephant, and Zebrafish. Therefore, we applied mouse model for further in-vivo research.

A

Matrix ID	Name	Score	Relative score	Sequence ID	Start	End	Strand	Predicted sequence
MA0071.1	MA0071.1.RORA	8.901667	0.8491634062173469	NC_000006.12:21433646-21435646	645	654	+	CTTGAGGTCA
MA0071.1	MA0071.1.RORA	8.901667	0.8491634062173469	NC_000006.12:21433646-21435646	1598	1607	+	CTTGAGGTCA
MA1531.1	MA1531.1.NR1D1	7.621619	0.846694034929422	NC_000006.12:21433646-21435646	649	663	+	AGGTCAGGAGTTCGA
MA0071.1	MA0071.1.RORA	8.438349	0.8363726976532859	NC_000006.12:21433646-21435646	85	94	+	AAAAAAGTCA
MA0071.1	MA0071.1.RORA	8.438349	0.8363726976532859	NC_000006.12:21433646-21435646	1151	1160	+	AAAAATGTCA
MA0071.1	MA0071.1.RORA	8.330314	0.8333901981508683	NC_000006.12:21433646-21435646	1530	1539	+	TACAAAGTCA
MA1532.2	MA1532.2.NR1D2	4.463795	0.8285371594253184	NC_000006.12:21433646-21435646	648	662	+	GAGGTCAGGAGTTCG
MA1531.1	MA1531.1.NR1D1	4.018153	0.8115419117993256	NC_000006.12:21433646-21435646	1498	1512	-	GTTTCACTATGTTAG
MA1532.2	MA1532.2.NR1D2	1.1565083	0.8012230373398895	NC_000006.12:21433646-21435646	1499	1513	-	GGTTTCACTATGTTA

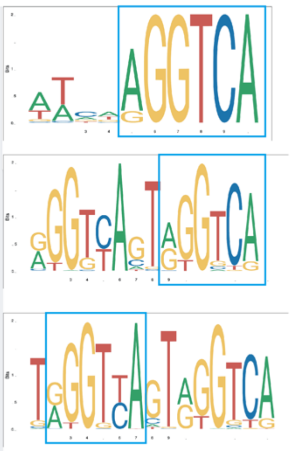

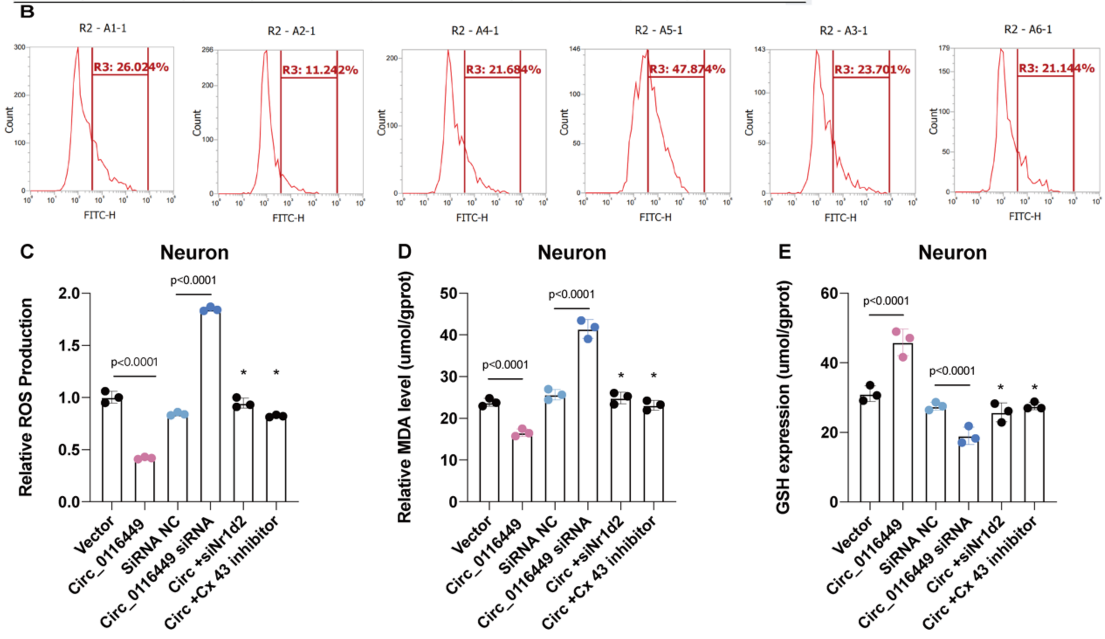

Supp Figure 3. The JASPAR dataset is used to predict the potential binding among RORA, NR1D1, NR1D2 and promoter of Cx43 (A). The relative ROS production, MDA level and GSH expression among these groups (N=3, in each group; ⁎, P<0.05, B-E)
